# The contribution of gestational age, area deprivation and mother’s country of birth to ethnic variations in infant mortality in England and Wales: A national cohort study using routinely collected data

**DOI:** 10.1371/journal.pone.0195146

**Published:** 2018-04-12

**Authors:** Yangmei Li, Maria A. Quigley, Nirupa Dattani, Ron Gray, Hiranthi Jayaweera, Jennifer J. Kurinczuk, Alison Macfarlane, Jennifer Hollowell

**Affiliations:** 1 Policy Research Unit in Maternal Health and Care, National Perinatal Epidemiology Unit, Nuffield Department of Population Health, University of Oxford, Oxford, United Kingdom; 2 Centre for Maternal and Child Health Research, School of Health Sciences, University of London, London, United Kingdom; 3 School of Anthropology, University of Oxford, Oxford, United Kingdom; Centre Hospitalier Universitaire Vaudois, FRANCE

## Abstract

**Objectives:**

We aimed to describe ethnic variations in infant mortality and explore the contribution of area deprivation, mother’s country of birth, and prematurity to these variations.

**Methods:**

We analyzed routine birth and death data on singleton live births (gestational age≥22 weeks) in England and Wales, 2006–2012. Infant mortality by ethnic group was analyzed using logistic regression with adjustment for sociodemographic characteristics and gestational age.

**Results:**

In the 4,634,932 births analyzed, crude infant mortality rates were higher in Pakistani, Black Caribbean, Black African, and Bangladeshi infants (6.92, 6.00, 5.17 and 4.40 per 1,000 live births, respectively vs. 2.87 in White British infants). Adjustment for maternal sociodemographic characteristics changed the results little. Further adjustment for gestational age strongly attenuated the risk in Black Caribbean (OR 1.02, 95% CI 0.89–1.17) and Black African infants (1.17, 1.06–1.29) but not in Pakistani (2.32, 2.15–2.50), Bangladeshi (1.47, 1.28–1.69), and Indian infants (1.24, 1.11–1.38). Ethnic variations in infant mortality differed significantly between term and preterm infants. At term, South Asian groups had higher risks which cannot be explained by sociodemographic characteristics. In preterm infants, adjustment for degree of prematurity (<28, 28–31, 32–33, 34–36 weeks) fully explained increased risks in Black but not Pakistani and Bangladeshi infants. Sensitivity analyses with further adjustment for small for gestational age, or excluding deaths due to congenital anomalies did not fully explain the excess risk in South Asian groups.

**Conclusions:**

Higher infant mortality in South Asian and Black infants does not appear to be explained by sociodemographic characteristics. Higher proportions of very premature infants appear to explain increased risks in Black infants but not in South Asian groups. Strategies targeting the prevention and management of preterm birth in Black groups and suboptimal birthweight and modifiable risk factors for congenital anomalies in South Asian groups might help reduce ethnic inequalities in infant mortality.

## Introduction

Marked ethnic disparities in infant mortality have been observed in many countries [1–6], including England and Wales [7,8] and present a major public health challenge. In general the reasons why certain ethnic groups have higher infant mortality rates are not well understood [2,9–12].

A number of individual risk factors for infant mortality are well established, including preterm birth, low birth weight, intrauterine growth restriction, and socioeconomic status [8,10,13–15]. Patterns of these risk factors have been shown to vary by ethnic group, and also within ethnic groups [16–18]. For example, in the UK, low birth weight is more common in South Asian groups, whereas preterm birth is more common in Black groups. Variation in these factors, together with other community-level factors such as access to care, have been suggested as potential reasons for ethnic differences in infant mortality [2,4,9,19–21].

Most previous analyses of ethnic variations in infant mortality in England and Wales have either used mother’s country of birth or mother’s/parents’ ethnic group(s) rather than infant’s ethnic group [22–26]. Additionally many studies have been conducted in small geographical regions that lack the ethnic diversity of the broader population of England and Wales [22,23,25–27]. Since 2005, national data on all births and infant deaths in England and Wales have been routinely linked to birth notification records which include data on infant’s ethnic group and gestational age [28,29]. Previous analysis of these national data for the years 2005 and 2006 using infant’s ethnic group generally found higher infant mortality rates in South Asian and Black minority ethnic groups compared with the White British group [7,8,30]. However, these studies only covered a relatively short period of time [30], reported only crude rates [7,30], or aggregated ethnic groups into larger groups (South Asian and Black) and hence did not provide information for smaller and more homogeneous ethnic groups [8]. Recent data are particularly important because of the upward trend in the number of births to women born outside the UK (27.5% of births in England and Wales in 2015 compared with 20.8% in 2005 and 12.6% in 1995) [31] and the changing patterns of migration. The number of births and deaths to infants in smaller ethnic groups is also now sufficiently large to have adequate power to analyze infant mortality subdivided into smaller minority ethnic groups.

The specific objectives of this study were (a) to describe ethnic variations in infant mortality in England and Wales; (b) to explore the individual and joint effects of infant’s ethnic group, area deprivation and mother’s country of birth, subdivided into UK and non-UK on infant mortality; and (c) to further explore the contribution of preterm birth to ethnic variations in infant mortality.

## Methods

### Study design, setting, data sources and study population

The study was based on a retrospective population-based cohort using routinely collected and linked national data on all singleton live births at gestational ages of 22 weeks and over in England and Wales from 2006 to 2012 inclusive.

Birth and death registration data are routinely collected and linked for all births and deaths in the first year of life in England and Wales. Since 2005, these data have been further linked with the National Health Service (NHS) number for babies (NN4B) electronic system. The NN4B system issues an NHS number to all births as soon as possible after birth and provides additional key birth details not collected at birth registration, such as gestational age at birth and the ethnic group of the infant [28,29]. All data were fully anonymized before being made available to the study researchers. The study was approved by ‘National Research Ethics Service (NRES) Committee South Central—Oxford B’ (Research Ethics Committee reference number: 15/SC/0493).

We cleaned the data extract provided by Office for National Statistics (ONS) by removing records with implausible or missing values in gestational age and birth weight. We sequentially excluded (a) births with an implausible gestational age, i.e. equal to or more than 43 weeks; (b) births with a missing birth weight; (c) births with an implausible birth weight for gestational age, defined as birth weight more than twice the interquartile range above or below the median, based on sex-gestation-ethnic group-specific birth weight centiles of the study cohort.

### Infant’s ethnic group and other explanatory variables

The ethnic group data used for this study are the infant’s ethnic group as reported in the NN4B birth notification system, guided by a ‘pick list’ with the ethnic categories used in the 2001 Census in England and Wales [16]. In practice it is unclear whether the ethnic group is the mother’s or the baby’s or whether it was reported by the mother or a health professional [16]. The approach used to develop the ethnic group categories used in the UK has been described elsewhere [32]. The categories used in this study are White British, Other White, three Asian or Asian British groups (Indian, Pakistani, and Bangladeshi), two Black or Black British groups (Black Caribbean and Black African), a ‘Mixed/Other’ group, which included all Mixed groups, Other Asian, Other Black, Chinese, and Other, and a ‘Not stated’ group, which consisted of infants whose ethnic group was not reported in the NN4B system. Details of the derivation of the ethnic group categories are given in S1 Table.

The main explanatory variables included area deprivation score of the mother’s area of residence (as measured by quintiles of the 2015 English Index of Multiple Deprivation (IMD) [33] and the 2014 Welsh IMD [34]), mother’s country of birth (UK versus non-UK), sex of infant, infant’s year of birth, age of mother in years (under 18, 18–19, 20–24, 25–29, 30–34, 35–39, 40 and over), marital status/registration type (married, joint registration/same address, joint registration/different address, sole registration), mother’s country of residence (England versus Wales), gestational age in weeks (under 28, 28–31, 32–33, 34–36, 37–38, 39–41, 42), and preterm birth (gestational age of 37 weeks or more versus gestational age of less than 37 weeks). The numerical variables we used, including infant’s year of birth, age of mother, and gestational age, did not exhibit a linear association with infant mortality; therefore they were categorized in the analysis as mentioned above. For one of the sensitivity analyses, we also considered small for gestational age (SGA), defined as a birth weight below the 10^th^ percentile for gestational age and sex derived from our study population.

### Outcome measures

The primary outcome was infant death, defined as death before one year of age following a live birth. Infant mortality rates were calculated as the number of infant deaths per 1,000 live births. In some analyses, we considered infant mortality by cause and used the ONS cause groups [35]. This is a hierarchical classification, based on the Wigglesworth classification system [36] which is designed to capture the underlying ‘mechanism’ which led to infant death. Infant deaths were classified into the following cause of death groups: 1. Congenital anomalies; 2. Antepartum infections; 3. Immaturity related conditions; 4. Asphyxia, anoxia, or trauma; 5. External conditions; 6. Infections; 7. Other specific conditions; 9. Sudden infant deaths; 0. Other conditions.

### Statistical analysis

Logistic regression models were used to explore the association between ethnic group and infant mortality. We adjusted all models for sex of infant and their year of birth to account for differences between sexes and temporal changes in infant mortality (models adjusted for these two variables only are referred to as the ‘base model’ hereafter). A series of sequential adjustments were made to explore the association between infant mortality and ethnic group, IMD quintile, and mother’s country of birth, both individually and jointly (objective b). First, these three exposure variables were added individually to the base model without mutual adjustment to explore the unadjusted associations between ethnic group, IMD quintile, mother’s country of birth and infant mortality (objective b). Second, we adjusted for all maternal sociodemographic characteristics, including IMD quintile, mother’s country of birth, age of mother and marital status/registration type (referred to as ‘model A’), to explore the joint effects of infant’s ethnic group, area deprivation and mother’s country of birth, taking account of other potential confounders (objective b). Finally, we additionally adjusted for gestational age (referred to as ‘model B’ or ‘fully adjusted model’ hereafter) to explore whether gestational age further explained ethnic variations in infant mortality (objective c). Because IMD scores are constructed differently in England and in Wales [37], we adjusted for mother’s country of residence (England versus Wales) in all models that included IMD.

To further explore the joint effect of ethnic group, IMD and mother’s country of birth, we tested the pairwise interaction between ethnic group and IMD, ethnic group and mother’s country of birth, IMD and mother’s country of birth, respectively using a Likelihood Ratio Test (LRT). We tested for an interaction between ethnic group and preterm birth using a LRT to further explore the contribution of preterm birth to ethnic variations in infant mortality.

We conducted a sensitivity analysis in which we additionally adjusted for SGA (referred to as ‘model C’ hereafter). Because infant mortality attributed to congenital anomalies is known to be higher in Pakistani infants [7], a separate sensitivity analysis excluding congenital anomalies from infant deaths was conducted to see whether this explained the higher infant mortality rates in the Pakistani group.

All analyses were conducted in STATA version 13 (Stata Corporation, College Station, USA) [38], with the White British group as the reference group. All p-values presented were two tailed and statistical significance was set at the 0.05 level, except for interaction tests where the statistical significance was set at the 0.01 level to allow for multiple testing.

## Results

The linked dataset included 4,744,666 singleton live births at gestational ages of 22 weeks or more in England and Wales from 2006 to 2012. In total, we excluded 109,734 (2.3%) births, which included births with implausible gestational age (n = 16,695), missing birth weight (n = 20,999), or implausible sex-gestation-ethnic group-specific birth weight (n = 72,040). The study population therefore consisted of 4,634,932 singleton live born infants at gestational ages of 22 weeks or more and not more than 42 weeks. Among them, approximately 65% were White British, 10% were South Asian (including Indian, Pakistani and Bangladeshi), 7% were Other White, 5% were Black (including Black Caribbean and Black African), 4% were of the Mixed groups, and 2.5% were of other ethnic backgrounds. The ethnic group of around 6% of the study population was ‘not stated’.

### Characteristics of the mothers and infants

The characteristics of the mothers and the infants varied by ethnic group (Table 1). Compared with White infants (White British and Other White), infants of other ethnic backgrounds were generally more likely to be born at lower gestational ages. Rates of very preterm birth were particularly high among infants of Black Caribbean and Black African origin. Around 50–60% of Pakistani, Bangladeshi, Black Caribbean and Black African infants were born to mothers living in the most deprived areas (IMD quintile 1) and around 80% were born to mothers living in the two most deprived areas (IMD quintiles 1 and 2 combined) while these proportions were around 23% and 44% respectively in White British infants. Only 4% of mothers of White British infants were born outside the UK, compared with 37% of mothers of Black Caribbean infants and 26% of mothers of infants whose ethnic group was ‘not stated’. For infants of other ethnic groups, the majority of their mothers were born outside the UK, ranging from 60% of the Mixed/Other group to 93% of Black African infants.

**Table 1:** Characteristics of the study population by ethnic group (percentage, singleton live births, England and Wales, 2006–2012).

Ethnic group	White British	Other White	Indian	Pakistani	Bangladeshi	Black Caribbean	Black African	Mixed / Other	Not stated
**Total number**	3,009,231	340,526	132,651	180,269	62,948	47,505	154,076	419,970	287,756
**Sex of infant**									
Male	51.3	51.5	51.3	51.0	50.7	50.8	50.6	51.4	51.4
Female	48.7	48.5	48.7	49.0	49.3	49.2	49.4	48.6	48.7
**Infant’s year of birth**									
2006	13.5	10.4	12.2	13.2	13.4	14.7	13.0	12.3	21.8
2007	13.7	12.3	12.8	13.7	14.0	14.6	13.5	13.2	20.3
2008	14.0	13.3	13.0	14.6	14.0	14.3	14.2	13.8	19.9
2009	14.3	14.1	14.4	14.4	14.4	14.7	14.4	14.3	13.6
2010	14.8	15.6	15.1	14.0	14.4	14.2	15.1	14.9	9.2
2011	14.9	16.5	16.1	14.7	14.8	13.7	14.8	15.3	7.4
2012	14.8	17.7	16.5	15.4	15.1	13.9	15.1	16.3	7.9
**Gestational age (completed weeks)**									
Under 28	0.3	0.2	0.3	0.4	0.3	0.9	0.7	0.4	0.4
28–31	0.6	0.4	0.6	0.7	0.5	1.1	0.9	0.6	0.6
32–33	0.7	0.5	0.7	0.7	0.7	1.0	0.8	0.7	0.7
34–36	4.0	3.5	4.4	4.3	4.7	5.2	3.8	4.0	4.0
37–38	17.4	17.1	24.4	22.7	25.9	22.5	20.0	20.2	18.3
39–41	72.9	74.2	67.2	68.9	65.6	66.7	69.3	70.8	72.0
42	4.1	4.0	2.4	2.4	2.3	2.6	4.5	3.4	4.1
**Age of mother, years**									
Under 18	2.0	0.7	0.1	0.2	0.3	2.5	0.6	1.5	1.6
18–19	5.1	2.0	0.6	1.7	1.9	6.0	1.9	3.8	4.2
20–24	20.0	15.0	12.2	22.9	25.5	22.3	13.9	18.3	18.0
25–29	25.8	29.7	36.6	37.6	37.0	25.6	29.3	27.6	26.5
30–34	27.3	31.8	35.6	25.2	24.5	21.5	31.4	28.1	28.6
35–39	16.2	17.1	12.7	10.2	9.1	15.2	17.6	16.5	17.1
40–44	3.5	3.5	2.0	2.0	1.5	6.4	4.8	4.1	3.7
45 and over	0.2	0.2	0.2	0.1	0.1	0.5	0.4	0.3	0.2
**Deprivation quintile**									
1 (most deprived)	23.4	23.8	24.3	55.3	59.8	49.8	50.1	34.2	22.0
2	20.2	26.1	29.9	25.1	24.8	29.9	29.6	25.9	23.1
3	19.5	20.0	20.8	10.8	8.8	13.0	11.9	17.2	20.1
4	18.9	16.2	13.6	5.4	4.3	4.8	5.4	12.5	18.6
5 (least deprived)	18.0	14.0	11.4	3.4	2.3	2.4	3.1	10.3	16.2
**Mother’s country of residence**									
England	93.4	97.8	98.3	99.1	97.8	99.7	99.0	97.2	97.4
Wales	6.6	2.2	1.7	0.9	2.2	0.3	1.0	2.8	2.6
**Mother’s country of birth**^a^									
UK	96.1	19.9	34.0	37.1	21.8	63.3	7.3	40.4	73.6
Non-UK	3.9	80.1	66.0	62.9	78.2	36.7	92.7	59.6	26.4
**Marital status/registration type**									
Married	46.2	64.8	96.2	95.7	94.6	26.5	61.3	61.5	57.1
Joint registration / same address	36.7	26.8	2.1	1.9	2.7	21.7	14.6	19.1	28.6
Joint registration / different address	10.9	4.0	0.9	1.1	1.6	32.0	12.4	11.2	8.3
Sole registration	6.2	4.4	0.9	1.3	1.2	19.8	11.8	8.2	6.1

^a^ Percentages based on study population excluding records with missing data on mother’s country of birth (n = 143 in total)

Compared with parents of White British infants, parents of Other White and South Asian infants were more likely to be married or jointly registered as living at the same address while parents of Black Caribbean and Black African infants were less likely to be married or jointly registered as living at the same address.

### Infant mortality by characteristics of the mothers and infants

In total, there were 15,001 infant deaths in the study population. The overall infant mortality rate was 3.24 per 1,000 live births in the whole study population and 2.87 in White British infants (Table 2). Crude infant mortality rates generally decreased by year and varied by characteristics of the mothers and the infants. Infants of all minority ethnic groups, except Other White, had higher crude infant mortality rates than White British infants. Infant mortality was inversely associated with gestational age and the lowest rates were observed in infants born at gestational ages of 39–41 weeks (1.19 per 1,000 live births). Infant mortality rates showed a U shape association with age of mother, with those born to mothers under 20 years or 45 years or over having the highest mortality and those born to mothers aged 30–34 years having the lowest mortality. Infants born to mothers living in the least deprived areas and those born to mothers who were married or jointly registered as living at the same address with their partner had lower infant mortality rates while infants born to mothers who were born outside the UK had higher crude mortality rates.

**Table 2:** Infant mortality by maternal and infants’ characteristics (singleton live births, England and Wales, 2006–2012).

Characteristics	Infant deaths	Total live births	Infant mortality rate		
			per 1,000 live births (95% CI)		
**All**	15,001	4,634,932	3.24	3.19	3.29
**Infant’s ethnic group**					
White British	8,634	3,009,231	2.87	2.81	2.93
Other White	838	340,526	2.46	2.30	2.63
Indian	473	132,651	3.57	3.26	3.90
Pakistani	1,247	180,269	6.92	6.55	7.31
Bangladeshi	277	62,948	4.40	3.91	4.95
Black Caribbean	285	47,505	6.00	5.34	6.74
Black African	797	154,076	5.17	4.83	5.54
Mixed/Other	1,431	419,970	3.41	3.24	3.59
Not stated	1,019	287,756	3.54	3.33	3.77
**Sex of infant**					
Male	8,465	2,377,766	3.56	3.49	3.64
Female	6,536	2,257,166	2.90	2.83	2.97
**Infant’s year of birth**					
2006	2,303	631,705	3.65	3.50	3.80
2007	2,383	646,902	3.68	3.54	3.83
2008	2,241	663,918	3.38	3.24	3.52
2009	2,130	659,807	3.23	3.09	3.37
2010	2,042	671,265	3.04	2.91	3.18
2011	2,033	675,075	3.01	2.88	3.15
2012	1,869	686,260	2.72	2.60	2.85
**Gestational age (completed weeks)**					
Under 28	5,078	13,999	362.74	354.81	370.74
28–31	1,354	26,694	50.72	48.15	53.42
32–33	595	31,506	18.89	17.44	20.45
34–36	1,498	186,316	8.04	7.64	8.46
37–38	2,275	851,555	2.67	2.56	2.78
39–41	3,981	3,344,894	1.19	1.15	1.23
42	220	179,968	1.22	1.07	1.40
**Age of mother, years**					
Under 18	423	76,687	5.52	5.02	6.07
18–19	962	199,193	4.83	4.53	5.14
20–24	3,331	886,858	3.76	3.63	3.89
25–29	3,975	1,265,724	3.14	3.04	3.24
30–34	3,527	1,295,682	2.72	2.63	2.81
35–39	2,118	738,929	2.87	2.75	2.99
40–44	623	163,080	3.82	3.53	4.13
45 and over	42	8,779	4.78	3.54	6.47
**Deprivation quintile**					
1 (most deprived)	5,566	1,261,026	4.41	4.30	4.53
2	3,665	1,031,727	3.55	3.44	3.67
3	2,408	862,178	2.79	2.68	2.91
4	1,834	771,250	2.38	2.27	2.49
5 (least deprived)	1,528	708,751	2.16	2.05	2.27
**Mother’s country of residence**					
England	14,237	4,402,845	3.23	3.18	3.29
Wales	764	232,087	3.29	3.07	3.53
**Mother’s country of birth**^a^					
UK	10,955	3,507,324	3.12	3.07	3.18
Non-UK	4,043	1,127,465	3.59	3.48	3.70
**Marital status/registration type**					
Married	7,210	2,499,063	2.89	2.82	2.95
Joint registration/same address	4,433	1,398,935	3.17	3.08	3.26
Joint registration/different address	2,001	450,500	4.44	4.25	4.64
Sole registration	1,357	286,434	4.74	4.49	5.00

^a^ Numbers based on study population excluding records with missing data on mother’s country of birth (n = 143 in total)

### Cause of infant deaths by ethnic group

**Fig 1:**
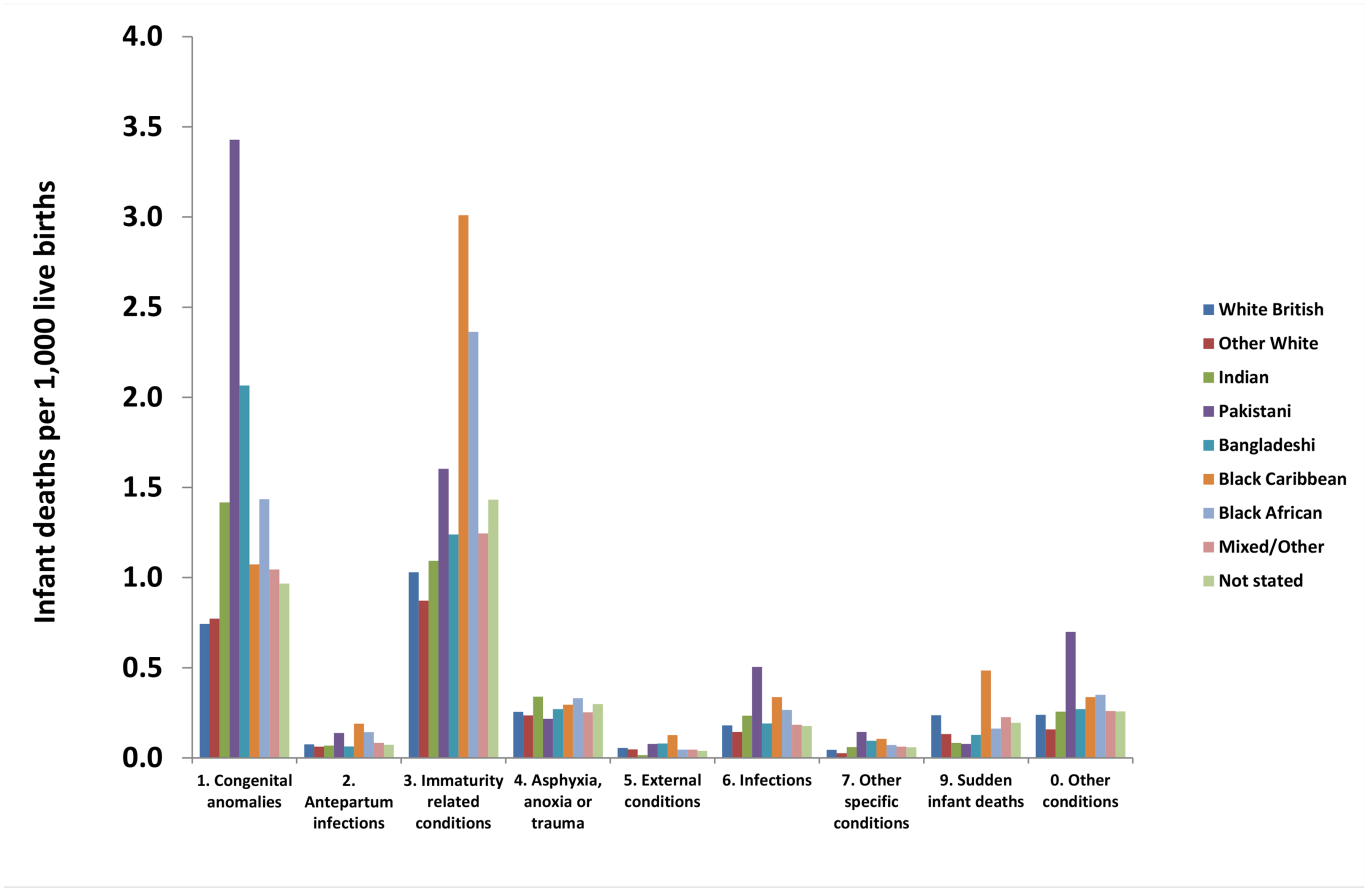
Cause of death by ethnic group (singleton live births, England and Wales, 2006–2012).

Fig 1 and S2 Table show the causes of infant deaths by ethnic group. The Pakistani group had the highest rate of infant death due to ‘congenital anomalies’ (3.43 per 1,000 live births (95%CI 3.17–3.71) compared with 0.74 per 1,000 live births (95%CI 0.71–0.77) in White British infants). Although much lower than in the Pakistani group, rates were also markedly high compared with the White British group in the other two South Asian groups and the Black African group. The rates were 2.07 per 1,000 live births (95%CI 1.74–2.45) for the Bangladeshi group, 1.42 per 1,000 live births (95%CI 1.23–1.63) for the Indian group, and 1.43 per 1,000 live births (95%CI 1.26–1.64) for the Black African group. The Black Caribbean and the Black African groups had the highest rates of infant deaths attributed to ‘immaturity related conditions’ with rates of 3.01 per 1,000 live births (95%CI 2.56–3.55) for the Black Caribbean group and 2.36 per 1,000 live births (95%CI 2.13–2.62) for the Black African group. Other causes of death also varied between ethnic groups: Black Caribbean infants had the highest rate of ‘sudden infant deaths’ (0.48 per 1,000 live births (95%CI 0.32–0.73) versus 0.24 per 1,000 live births (95%CI 0.22–0.25) for White British infants) and Pakistani infants had a higher rate of deaths attributed to ‘infections’ than White British infants (0.50 per 1,000 live births (95%CI 0.41–0.62) versus 0.18 per 1,000 live births (95%CI 0.17–0.20) respectively).

### Individual and joint effects of ethnic group, area deprivation and mother’s country of birth

Infants of all non-White ethnic groups had higher infant mortality rates compared with White British infants (ORs between 1.19–2.42) in the base model (adjusted only for infant’s sex and year of birth) with Pakistani and Black Caribbean infants having the highest mortality (Table 3). The lowest rate was observed in Other White infants (OR 0.87, 95%CI 0.81–0.93) in the base model. Both IMD and mother’s country of birth were associated with infant mortality in the base model, with a decreasing trend towards IMD quintile 5 (least deprived) and infants of mothers born outside the UK having higher infant mortality rates in the base model.

**Table 3:** Unadjusted and adjusted associations between ethnic group, area deprivation, mother’s country of birth and infant mortality (singleton live births, England and Wales, 2006–2012).

	Base model^a^			Model A^b^			Model B^c^		
	OR	(95% CI)		OR	(95% CI)		OR	(95% CI)	
**Infant’s ethnic group**									
White British	1	-		1	-		1	-	
Other White	0.87	0.81	0.93	0.98	0.91	1.06	0.99	0.91	1.08
Indian	1.25	1.14	1.37	1.51	1.37	1.67	1.24	1.11	1.38
Pakistani	2.42	2.28	2.57	2.61	2.43	2.80	2.32	2.15	2.50
Bangladeshi	1.54	1.36	1.73	1.65	1.45	1.87	1.47	1.28	1.69
Black Caribbean	2.09	1.86	2.35	1.69	1.50	1.91	1.02	0.89	1.17
Black African	1.81	1.69	1.95	1.78	1.63	1.94	1.17	1.06	1.29
Mixed/Other	1.19	1.13	1.26	1.21	1.14	1.29	1.06	0.99	1.13
Not stated	1.19	1.11	1.27	1.25	1.17	1.33	1.12	1.04	1.20
**Deprivation quintile**									
1 (most deprived)	2.06	1.95	2.18	1.59	1.50	1.69	1.35	1.26	1.43
2	1.66	1.56	1.76	1.41	1.32	1.50	1.25	1.17	1.33
3	1.30	1.22	1.39	1.20	1.12	1.28	1.12	1.04	1.20
4	1.11	1.03	1.18	1.07	1.00	1.14	1.02	0.95	1.10
5 (least deprived)	1	-		1	-		1	-	
**Mother’s country of birth**									
UK	1	-		1	-		1	-	
Non-UK	1.16	1.12	1.20	0.92	0.88	0.97	0.97	0.92	1.02

^a^ Base model adjusted for sex of infant and infant’s year of birth only

^b^ Model A adjusted for variables in Base model and additionally adjusted for age of mother, deprivation quintile, mother’s country of birth (UK vs. non-UK) and marital status/registration type

^c^ Model B adjusted for variables in Model A and additionally adjusted for gestational age in completed weeks (under 28, 28–31, 32–33, 34–36, 37–38, 39–41, 42)

However, after further adjustment for age of mother, IMD quintile, mother’s country of birth, and marital status/registration type, the infant mortality rate was significantly lower for infants of mothers born outside the UK compared with infants of mothers born in the UK (OR 0.92, 95%CI 0.88–0.97). The magnitude of the association between IMD and infant mortality decreased after adjustment but a decreasing gradient was still observed, i.e. lower rates in infants with mothers living in the less deprived areas. This multivariable adjustment changed the association between infant’s ethnic group and infant mortality in different directions for different ethnic groups; the odds ratios increased in Indian, Pakistani, and Bangladeshi infants but decreased in Black Caribbean and Black African infants compared with the base model. After this multivariable adjustment, Other White infants no longer had a lower infant mortality rate than the White British infants (OR 0.98, 95%CI 0.91–1.06).

Further adjustment for gestational age (model B, fully adjusted model) attenuated the association between ethnic group and infant mortality in all ethnic groups, especially in the Black Caribbean and Black African infants. The odds of infant death for Black Caribbean infants were no longer increased compared with White British infants (OR 1.02, 95%CI 0.89–1.17) and the OR for Black African infants reduced to 1.17 (95%CI 1.06–1.29) in the fully adjusted model. The three South Asian groups all still had higher odds of infant death than White British infants, with a particularly high OR of 2.32 (95%CI 2.15–2.50) for Pakistani infants, and ORs of 1.24 (95%CI 1.11–1.38) for Indian infants and 1.47 (95%CI 1.28–1.69) for Bangladeshi infants. In this fully adjusted model, IMD remained inversely associated with infant mortality although the effect was attenuated whereas mother’s country of birth was no longer statistically significantly associated with infant mortality (Table 3). S3 Table shows the ORs for all covariates in the adjusted models.

We found no significant interactions (results not shown) between infant’s ethnic group and mother’s country of birth, between infant’s ethnic group and IMD, or between IMD and mother’s country of birth.

### Term vs. preterm stratified analysis

There was a significant interaction between gestational age dichotomized into term and preterm and infant’s ethnic group (LRT p<0.001). We therefore stratified the study population into term and preterm births to further investigate how the association between ethnic group and infant mortality differed between these groups (Table 4). To account for any residual differences in gestational age distribution between the ethnic groups, we further adjusted for categorical gestational age within these strata (37–38, 39–41, 42 weeks for term infants and under 28, 28–31, 32–33, 34–36 weeks for preterm infants). In term infants, the three South Asian groups all had more than 50% higher odds of infant death (model A) with a particularly high OR (2.87, 95%CI 2.59–3.17) in Pakistani infants compared with White British infants, while other ethnic groups had similar or only modestly increased odds (ORs≤1.3) compared with the White British group. Further adjustment for categorical gestational age (37–38 weeks, 39–41 weeks, and 42 weeks) in term infants did not materially change the results (model B). In preterm infants, increased odds in Bangladeshi and Indian infants compared with White British infants were modest (ORs<1.3, model A). In contrast, the odds in the Pakistani group was twice the odds in the White British (OR 2.01, 95%CI 1.82–2.23), closely followed by Black African (OR 1.97, 95%CI 1.76–2.20) and Black Caribbean (OR 1.65, 95%CI 1.42–1.92) groups. Further adjustment for degree of prematurity (<28 weeks, 28–31 weeks, 32–33 weeks, and 34–36 weeks) significantly attenuated the results in preterm Black infants but not in preterm South Asian infants (model B). Preterm Black Caribbean (OR 0.95, 95%CI 0.80–1.12) and Black African infants (OR 1.08, 95%CI 0.95–1.23) no longer had higher odds while preterm Pakistani (OR 1.88, 95%CI 1.68–2.11) and Bangladeshi infants (OR 1.39, 95%CI 1.14–1.69) still had higher odds compared with preterm White British infants.

**Table 4:** The association between ethnic group and infant mortality stratified by gestational age (singleton live births, England and Wales, 2006–2012).

Infant’s ethnic group	Live births	Infant deaths	Infant mortality rate			Base model^a^			Model A^a^^,^ ^b^			Model B^a^^,^ ^b^^,^ ^c^			Model C^a^^,^ ^b^^,^ ^c^^,^ ^d^		
	N	n	per 1,000 live births (95% CI)			OR	(95% CI)		OR	(95% CI)		OR	(95% CI)		OR	(95% CI)	
**Term infants**																	
White British	2,842,568	3,751	1.32	1.28	1.36	1	-		1	-		1	-		1	-	
Other White	324,779	346	1.07	0.96	1.18	0.82	0.73	0.91	0.90	0.79	1.02	0.90	0.80	1.02	0.91	0.80	1.03
Indian	124,667	222	1.78	1.56	2.03	1.36	1.19	1.56	1.54	1.33	1.79	1.44	1.25	1.67	1.19	1.03	1.38
Pakistani	169,456	636	3.75	3.47	4.06	2.86	2.63	3.11	2.87	2.59	3.17	2.73	2.47	3.03	2.35	2.12	2.60
Bangladeshi	58,984	131	2.22	1.87	2.64	1.69	1.42	2.01	1.68	1.39	2.02	1.55	1.29	1.87	1.28	1.06	1.55
Black Caribbean	43,605	87	2.00	1.62	2.46	1.51	1.22	1.86	1.23	0.99	1.53	1.19	0.96	1.47	1.11	0.89	1.38
Black African	144,542	257	1.78	1.57	2.01	1.35	1.19	1.53	1.30	1.12	1.50	1.29	1.11	1.49	1.25	1.08	1.44
Mixed/Other	396,292	619	1.56	1.44	1.69	1.19	1.09	1.30	1.18	1.08	1.30	1.16	1.05	1.27	1.11	1.01	1.22
Not stated	271,524	427	1.57	1.43	1.73	1.14	1.03	1.27	1.19	1.07	1.31	1.18	1.07	1.31	1.15	1.04	1.28
**Preterm infants**																	
White British	166,663	4883	29.30	28.50	30.12	1	-		1	-		1	-		1	-	
Other White	15,747	492	31.24	28.64	34.08	1.07	0.98	1.18	1.11	1.00	1.23	1.11	0.98	1.25	1.12	0.99	1.27
Indian	7,984	251	31.44	27.83	35.50	1.08	0.95	1.23	1.17	1.02	1.34	1.06	0.91	1.23	0.98	0.84	1.15
Pakistani	10,813	611	56.51	52.31	61.02	1.99	1.82	2.16	2.01	1.82	2.23	1.88	1.68	2.11	1.77	1.58	1.99
Bangladeshi	3,964	146	36.83	31.40	43.17	1.27	1.07	1.50	1.27	1.06	1.52	1.39	1.14	1.69	1.30	1.07	1.59
Black Caribbean	3,900	198	50.77	44.30	58.12	1.77	1.53	2.05	1.65	1.42	1.92	0.95	0.80	1.12	0.92	0.77	1.09
Black African	9,534	540	56.64	52.17	61.46	2.00	1.82	2.19	1.97	1.76	2.20	1.08	0.95	1.23	1.05	0.93	1.20
Mixed/Other	23,678	812	34.29	32.05	36.69	1.18	1.09	1.27	1.18	1.08	1.28	0.98	0.89	1.07	0.97	0.88	1.07
Not stated	16,232	592	36.47	33.69	39.47	1.22	1.12	1.34	1.25	1.14	1.37	1.06	0.96	1.17	1.05	0.95	1.16

^a^ Adjusted for sex of infant and infant’s year of birth

^b^ Additionally adjusted for age of mother, deprivation quintile, mother’s country of birth (UK vs. non-UK) and marital status/registration type

^c^ Additionally adjusted for gestational age in completed weeks (37–38, 39–41, 42 for term infants and under 28, 28–31, 32–33, 34–36 for preterm infants)

^d^ Additionally adjusted for small for gestational age as a sensitivity analysis

### Sensitivity analyses

In term infants, additional adjustment for SGA (model C) further reduced the odds in all three South Asian groups compared with model B (OR 1.19, 95%CI 1.03–1.38 for Indian, OR 2.35, 95%CI 2.12–2.60 for Pakistani, OR 1.28, 95%CI 1.06–1.55 for Bangladeshi infants), but all three groups still had higher odds of infant death compared with the term White British (full results shown in Table 4). In preterm infants, further adjustment for SGA (model C) only slightly reduced the odds in Pakistani and Bangladeshi groups compared with model B.

In the sensitivity analysis of infant mortality from all causes other than congenital anomalies, the odds of death in Pakistani infants remained higher than in White British infants in both term (OR 1.93, 95%CI 1.66–2.25) and preterm infants (OR 1.38, 95%CI 1.20–1.58). Term Indian infants also still had higher odds of infant death from all causes other than congenital anomalies compared with term White British infants (complete results shown in S4 Table).

## Discussion

### Main findings

There are marked ethnic differences in infant mortality rates for singleton live births in England and Wales. Crude infant mortality rates are highest in Pakistani, Black Caribbean, Black African, and, to a lesser extent Bangladeshi infants. Adjustment for maternal sociodemographic characteristics including area deprivation and mother’s country of birth does not fundamentally change the pattern. Further adjustment for gestational age, however, significantly attenuates the risk of infant death in Black Caribbean and Black African infants but not in Pakistani, Bangladeshi, and Indian infants.

The association between ethnic group and infant mortality differs significantly between term and preterm infants. In term infants, South Asian groups (Pakistani, Bangladeshi, and Indian) all have higher risks of death which are not explained by maternal sociodemographic characteristics. In preterm infants, adjustment for degree of prematurity fully explains the higher risks of infant death in preterm Black Caribbean and Black African infants compared with White British infants but not in preterm Pakistani and Bangladeshi infants, who have lower rates of very preterm birth. Additional adjustment for SGA appears to further explain some but not all of the residual excess risks in the South Asian groups. Furthermore, the higher rate of death due to congenital anomalies in Pakistani infants does not appear to explain all the excess risk of infant mortality in this group.

### Strengths and weaknesses of the study

Major strengths of our study are that the large sample size has given us the power to study smaller more homogeneous ethnic subgroups rather than broad grouping such as ‘South Asians’; and that we were able to study infant’s ethnic group directly. Additionally the use of statutorily collected, linked birth and death registration data with cohort study design and low levels of missing data minimized potential biases such as selection bias, detection or observer bias and losses to follow-up, although we acknowledge that there may have been some losses to follow-up due to out migration.

The comprehensive national coverage of the study also means the findings reflect ethnic disparities in infant mortality across England and Wales. The generalizability of our findings to populations in countries other than England and Wales is likely to depend on the extent to which the ethnic groups are comparable to those in the UK and to other local factors such as disparities in access to healthcare.

Some misclassification of ethnic group seems possible, but it seems unlikely that this would have led to any systematic biases in our data except possibly in the mixed ethnic group where there is some limited data suggesting higher levels of misclassification [16]. This is a relatively small group which was not the focus of our study so any misclassification should have a minimal impact on our findings. Overall, ethnic group was not reported for 6.2% of records but this varied from 9.9% in 2006 through to 3.3% in 2012. We adjusted for infant’s year of birth in all analyses so temporal changes should not affect our findings. Previous analysis has suggested that this group is largely White and the characteristics of this group are similar to those of the White British group [16].

We lacked information on potentially important modifiable risk factors such as smoking, alcohol use, drug use, diet, and physical activity, maternal obesity, breastfeeding, and uptake of antenatal care.

The use of an area-based measure of deprivation (IMD) rather than an individual measure may be subject to the usual limitations of ecological summary measures [39]. However, IMD is available for all births and therefore was preferred over the National Statistics Socio-economic Classification, an individual socio-economic measure based on parents’ occupations only available for a 10 percent sample of live births.

### Comparison with previous studies and clinical implications

Previous studies and routinely published data for England and Wales from the 1980s onwards have generally found that South Asian, Black Caribbean and Black African infants tend to have higher infant mortality rates than White infants [7,8,22–27,30]. Although most of the studies reported mortality in the 1980s and the 1990s, our more recent findings are broadly consistent in that there are ethnic inequalities in infant mortality in England and Wales and the Pakistani and Black Caribbean infants continue to have the highest crude infant mortality rates.

An important, new finding of our study is that the factors that may explain relatively high infant mortality rates compared with the White British differ for Black and South Asian groups. Gestational age appears to explain almost all the elevated risks in the Black Caribbean and Black African infants but not in the South Asian groups. Higher rates of preterm birth, especially moderately preterm and very preterm birth in the Black groups have been well documented in England and Wales, in other high-income countries and worldwide [16,17,40–42]. Various causes of this excess in the Black groups have been suggested including an increased susceptibility to genital tract infections [41,43], which have been found to be more prevalent in Black women in the UK and elsewhere [41,44,45]. We found a larger proportion of infants born at very low gestational ages in the Black Caribbean and Black African groups in our cohort. The role of preterm birth is further supported by our analysis of cause of death which demonstrated a higher rate of infant deaths attributed to ‘immaturity related conditions’ in the Black Caribbean and Black African groups, consistent with previous national studies from England and Wales [7,24] and findings from the USA relating to Black American infants [19]. As Black Caribbean and Black American women are predominantly descended from people from West Africa, the possibility of a genetic component cannot be ignored. Other studies have also suggested that genetics might be an important factor that can partially explain ethnic disparities in infant mortality [2,12,46,47]. In this study, we did not disaggregate the Black African groups. A study of low birth weight in Black Caribbean and Black African infants, showed that when the Black African group was disaggregated by mother’s country of birth grouped into United Nation population regions, infants of mothers born in West Africa and Middle Africa but not those born in other parts of Africa have higher rates of preterm and very preterm birth than infants of mothers born in the UK [18].

The other novel finding of our study, the identification of an interaction between preterm birth and ethnic group has further demonstrated the importance of gestational age in the association between ethnic group and infant mortality, especially in explaining higher infant mortality in the Black groups. Further adjustment for degree of prematurity only appeared to explain the higher risks in preterm Black infants. This is broadly consistent with studies that show Black infants have similar or even lower gestational age-specific perinatal or infant mortality rates compared with the White infants only at very low gestational ages but not thereafter [23,48–50] possibly because of earlier fetal maturity [49,51].

On the other hand, neither preterm birth nor area deprivation, together with other maternal sociodemographic characteristics, explained the increased risks observed in the South Asian groups, in particular the Pakistani group. International studies also show that Pakistani infants have one of the highest infant mortality rates in other host countries [5,6]. Congenital anomalies are the most common cause of death amongst Pakistani infants [7,22], and risk of infant deaths due to ‘congenital anomalies’ was higher in the Pakistani group than other ethnic groups in our analysis. Consanguinity is more prevalent than in other ethnic groups [52] and differences appears to exist in provision or uptake of antenatal screening and prenatal diagnostic testing [53] and attitudes towards termination [54,55]. However, sensitivity analysis from our study showed that even when we excluded all deaths where the recorded cause of death was congenital anomaly, Pakistani infants still had a higher rate of infant death than White British infants.

Our study only looked at live births, but the finding of a higher risk of infant mortality in South Asian infants, whether term or preterm, is consistent with the patterns found by Balchin and colleagues that perinatal mortality rate (stillbirths plus early neonatal deaths per 1,000 births) was highest in infants of South Asian women at all gestational ages [56]. They also found that the increase in gestational age-specific perinatal mortality from term onwards was earliest and steepest in South Asian women, which may explain our findings that the risks of infant death at term were particularly high in South Asian groups.

Our exploratory analyses adjusting for SGA using cut-offs derived from our own population suggested that differences in SGA or birth weight between ethnic groups might explain some but not all of the residual excess risks in South Asian groups. Low birth weight is an established risk factor for infant mortality [57] but further research is needed to determine the most appropriate methods to identify pathologically small infants at risk of adverse outcomes in ethnically diverse populations.

Although the association between ethnic group and infant mortality in all the three South Asian groups changed in similar ways when adjusted for maternal sociodemographic characteristics and gestational age, there were still differences in infant mortality rates between the three groups, with Pakistani infants having particularly high risks and Indian and Bangladeshi infants having relatively lower and similar risks. Our data showed that fewer Indian than Pakistani and Bangladeshi infants had mothers living in deprived areas, however, this does not explain the much higher risks in Pakistani infants compared with Bangladeshi infants, nor the relatively small differences between Indian and Bangladeshi infants.

Various explanations have been proposed and discussed elsewhere which might explain the differences that we observed between South Asian groups, including genetics, health-related behavior and access to healthcare [12], but we lacked data to investigate these further.

### Implications for policy and practice

The identification of differing underlying factors for increased risks in mortality in South Asian and Black infants suggests that different prevention strategies may be required for these groups. The findings that gestational age, particularly more extreme preterm birth, appears to explain the increased risks in preterm Black infants, suggest a need for strategies to ensure that Black mothers have access to services for the prevention and management of preterm birth and for the conditions leading to iatrogenic preterm delivery. For South Asian groups, strategies targeting preventable risk factors for congenital anomalies and also ensuring access to antenatal screening for at risk groups, together with intervention strategies aimed at optimizing birth weight, may reduce some of the excess mortality, but further research is required to better understand other potentially preventable causes of excess mortality in these groups.

## Conclusions

In conclusion, South Asian and Black infants have higher infant mortality rates compared with White British infants which are not wholly explained by area deprivation, mother’s country of birth or other maternal characteristics. A higher proportion of infants born at lower gestational ages appears to explain the increased risks in preterm Black Caribbean and Black African infants but not in the South Asian groups. An increased risk of infant mortality due to congenital anomalies explains some but not all of the excess risk of infant mortality in South Asian, particularly Pakistani infants.

Our findings suggest that further research is needed into the causes of preterm birth in Black Caribbean and Black African mothers with the aim of developing strategies for the prevention and management of preterm birth and the causes of iatrogenic preterm delivery in these mothers and their infants. Excess risks in each of the three South Asian groups and the differences between these groups require further investigation, but strategies targeting suboptimal birth weight and modifiable risk factors for congenital anomalies in South Asian groups would be merited.

## Supporting information

S1 Table Ethnic group categories. (DOCX)

S2 Table Infant mortality rates by cause of death and ethnic group (per 1,000 live births, singleton live births, England and Wales, 2006–2012). (DOCX)

S3 Table The association between ethnic group and infant mortality (singleton live births, England and Wales, 2006–2012, full results for covariates in the adjusted models). (DOCX)

S4 Table The association between ethnic group and infant mortality excluding congenital anomalies and stratified by gestational age (singleton live births, England and Wales, 2006–2012). (DOCX)
