# Supplementary material for: The contribution of gestational age, area deprivation and mother’s country of birth to ethnic variations in infant mortality in England and Wales: A national cohort study using routinely collected data
Source: PLoS One. 2018 Apr 12;13(4):e0195146. doi: 10.1371/journal.pone.0195146 (PMC5896919; doi:10.1371/journal.pone.0195146)
Supplement: S1 Table — (DOCX) [file pone.0195146.s001.docx]

**Article title:**

## The contribution of gestational age, area deprivation and mother’s country of birth to ethnic variations in infant mortality in England and Wales: a national cohort study using routinely collected data

**Journal name:**

## Plos One

**Author names and affiliations:**

## Yangmei Li^1*^, Maria A. Quigley^1^, Nirupa Dattani^2^, Ron Gray^1^, Hiranthi Jayaweera^3^, Jennifer J. Kurinczuk^1^, Alison Macfarlane^2^, Jennifer Hollowell^1^

^1^ Policy Research Unit in Maternal Health and Care, National Perinatal Epidemiology Unit, Nuffield Department of Population Health, University of Oxford, Oxford, United Kingdom

^2^ Centre for Maternal and Child Health Research, School of Health Sciences, City, University of London, London, United Kingdom

^3^ School of Anthropology, University of Oxford, Oxford, United Kingdom

^*^ Correspondence author

E-mail: [yangmei.li@npeu.ox.ac.uk](mailto:yangmei.li@npeu.ox.ac.uk) (YL)

**Supplementary Table 1 (S1 Table) Ethnic group categories^a^**

| **Categories used in this study** | **Included ethnic groups** |
| --- | --- |
| 1 White British | British, Mixed British |
|  | English |
|  | Scottish |
|  | Welsh |
|  | Northern Irish |
|  | Cornish |
| 2 Other White | Irish |
|  | Any other White Background |
|  | Other white, white unspecified |
|  | Cypriot (part not stated) |
|  | Greek |
|  | Greek Cypriot |
|  | Turkish |
|  | Turkish Cypriot |
|  | Italian |
|  | Irish Traveler |
|  | Traveler |
|  | Gypsy/Romany |
|  | Polish |
|  | All republics which made up the former USSR |
|  | Kosovan |
|  | Albanian |
|  | Bosnian |
|  | Croatian |
|  | Serbian |
|  | Other republics which made up the former Yugoslavia |
|  | Mixed White |
|  | Other white European, European unspecified, European mixed |
| 3 Indian | Indian or British Indian |
| 4 Pakistani | Pakistani or British Pakistani |
| 5 Bangladeshi | Bangladeshi or British Bangladeshi |
| 6 Black Caribbean | Caribbean |
| 7 Black African | African |
|  | Somali |
|  | Nigerian |
| 8 Mixed/Other | White and Black Caribbean |
|  | White and Black African |
|  | White and Asian |
|  | Any other mixed background |
|  | Black and Asian |
|  | Black and Chinese |
|  | Black and White |
|  | Chinese and White |
|  | Asian and Chinese |
|  | Other mixed, mixed unspecified |
|  | Any other Asian background |
|  | Mixed Asian |
|  | Punjabi |
|  | Kashmiri |
|  | East African Asian |
|  | Sri Lanka |
|  | Tamil |
|  | Sinhalese |
|  | British Asian |
|  | Caribbean Asian |
|  | Other Asian, Asian unspecified |
|  | Any other Black background |
|  | Mixed Black |
|  | Black British |
|  | Other Black, Black unspecified |
|  | Chinese |
|  | Any other ethnic group |
|  | Vietnamese |
|  | Japanese |
|  | Filipino |
|  | Malaysian |
|  | Any other Group |
| 9 Not stated | Not Stated |

^a^ The classification was applied to the ONS variable ‘ethcatz’. Requests for the Stata code to generate these categories should be addressed to the corresponding author.
