# Supplementary material for: The contribution of gestational age, area deprivation and mother’s country of birth to ethnic variations in infant mortality in England and Wales: A national cohort study using routinely collected data
Source: PLoS One. 2018 Apr 12;13(4):e0195146. doi: 10.1371/journal.pone.0195146 (PMC5896919; doi:10.1371/journal.pone.0195146)
Supplement: S2 Table — (DOCX) [file pone.0195146.s002.docx]

**Article title:**

## The contribution of gestational age, area deprivation and mother’s country of birth to ethnic variations in infant mortality in England and Wales: a national cohort study using routinely collected data

**Journal name:**

## Plos One

**Author names and affiliations:**

## Yangmei Li^1*^, Maria A. Quigley^1^, Nirupa Dattani^2^, Ron Gray^1^, Hiranthi Jayaweera^3^, Jennifer J. Kurinczuk^1^, Alison Macfarlane^2^, Jennifer Hollowell^1^

^1^ Policy Research Unit in Maternal Health and Care, National Perinatal Epidemiology Unit, Nuffield Department of Population Health, University of Oxford, Oxford, United Kingdom

^2^ Centre for Maternal and Child Health Research, School of Health Sciences, City, University of London, London, United Kingdom

^3^ School of Anthropology, University of Oxford, Oxford, United Kingdom

^*^ Correspondence author

E-mail: [yangmei.li@npeu.ox.ac.uk](mailto:yangmei.li@npeu.ox.ac.uk) (YL)

**Supplementary Table 2 (S2 Table) Infant mortality rates by cause of death and ethnic group (per 1,000 live births, singleton live births, England and Wales, 2006-2012)**

| **Infant’s ethnic group** | **Congenital anomalies** | | **Antepartum infections** | | **Immaturity related conditions** | | **Asphyxia, anoxia or trauma** | | **External conditions** | |
| --- | --- | --- | --- | --- | --- | --- | --- | --- | --- | --- |
|  | rate | 95%CI | rate | 95%CI | rate | 95%CI | rate | 95%CI | rate | 95%CI |
| **White British** | 0.74 | (0.71-0.77) | 0.08 | (0.07-0.09) | 1.03 | (0.99-1.07) | 0.25 | (0.24-0.27) | 0.05 | (0.05-0.06) |
| **Other White** | 0.77 | (0.68-0.87) | 0.06 | (0.04-0.09) | 0.87 | (0.78-0.98) | 0.23 | (0.19-0.29) | 0.05 | (0.03-0.08) |
| **Indian** | 1.42 | (1.23-1.63) | 0.07 | (0.04-0.13) | 1.09 | (0.93-1.29) | 0.34 | (0.25-0.45) | 0.02 | (0.00-0.06) |
| **Pakistani** | 3.43 | (3.17-3.71) | 0.14 | (0.09-0.21) | 1.60 | (1.43-1.80) | 0.22 | (0.16-0.30) | 0.08 | (0.05-0.13) |
| **Bangladeshi** | 2.07 | (1.74-2.45) | 0.06 | (0.02-0.17) | 1.24 | (0.99-1.55) | 0.27 | (0.17-0.43) | 0.08 | (0.03-0.19) |
| **Black Caribbean** | 1.07 | (0.82-1.41) | 0.19 | (0.10-0.36) | 3.01 | (2.56-3.55) | 0.29 | (0.17-0.50) | 0.13 | (0.06-0.28) |
| **Black African** | 1.43 | (1.26-1.64) | 0.14 | (0.09-0.22) | 2.36 | (2.13-2.62) | 0.33 | (0.25-0.44) | 0.05 | (0.02-0.10) |
| **Mixed/Other** | 1.05 | (0.95-1.15) | 0.08 | (0.06-0.12) | 1.25 | (1.14-1.36) | 0.25 | (0.21-0.31) | 0.05 | (0.03-0.07) |
| **Not stated** | 0.97 | (0.86-1.09) | 0.07 | (0.05-0.11) | 1.43 | (1.30-1.58) | 0.30 | (0.24-0.37) | 0.04 | (0.02-0.07) |

(continued)

|  | **Infections** | | **Other specific conditions** | | **Sudden infant deaths** | | **Other conditions** | |
| --- | --- | --- | --- | --- | --- | --- | --- | --- |
|  | rate | 95%CI | rate | 95%CI | rate | 95%CI | rate | 95%CI |
| **White British** | 0.18 | (0.17-0.20) | 0.04 | (0.04-0.05) | 0.24 | (0.22-0.25) | 0.24 | (0.22-0.26) |
| **Other White** | 0.14 | (0.11-0.19) | 0.03 | (0.01-0.05) | 0.13 | (0.10-0.18) | 0.16 | (0.12-0.21) |
| **Indian** | 0.23 | (0.16-0.33) | 0.06 | (0.03-0.12) | 0.08 | (0.05-0.15) | 0.26 | (0.18-0.36) |
| **Pakistani** | 0.50 | (0.41-0.62) | 0.14 | (0.10-0.21) | 0.08 | (0.05-0.13) | 0.70 | (0.59-0.83) |
| **Bangladeshi** | 0.19 | (0.11-0.34) | 0.10 | (0.04-0.21) | 0.13 | (0.06-0.25) | 0.27 | (0.17-0.43) |
| **Black Caribbean** | 0.34 | (0.21-0.55) | 0.11 | (0.04-0.25) | 0.48 | (0.32-0.73) | 0.34 | (0.21-0.55) |
| **Black African** | 0.27 | (0.20-0.36) | 0.07 | (0.04-0.13) | 0.16 | (0.11-0.24) | 0.35 | (0.27-0.46) |
| **Mixed/Other** | 0.18 | (0.15-0.23) | 0.06 | (0.04-0.09) | 0.23 | (0.19-0.28) | 0.26 | (0.22-0.31) |
| **Not stated** | 0.18 | (0.13-0.23) | 0.06 | (0.04-0.10) | 0.19 | (0.15-0.25) | 0.26 | (0.20-0.32) |
