# Supplementary material for: The contribution of gestational age, area deprivation and mother’s country of birth to ethnic variations in infant mortality in England and Wales: A national cohort study using routinely collected data
Source: PLoS One. 2018 Apr 12;13(4):e0195146. doi: 10.1371/journal.pone.0195146 (PMC5896919; doi:10.1371/journal.pone.0195146)
Supplement: S3 Table — (DOCX) [file pone.0195146.s003.docx]

**Article title:**

## The contribution of gestational age, area deprivation and mother’s country of birth to ethnic variations in infant mortality in England and Wales: a national cohort study using routinely collected data

**Journal name:**

## Plos One

**Author names and affiliations:**

## Yangmei Li^1*^, Maria A. Quigley^1^, Nirupa Dattani^2^, Ron Gray^1^, Hiranthi Jayaweera^3^, Jennifer J. Kurinczuk^1^, Alison Macfarlane^2^, Jennifer Hollowell^1^

^1^ Policy Research Unit in Maternal Health and Care, National Perinatal Epidemiology Unit, Nuffield Department of Population Health, University of Oxford, Oxford, United Kingdom

^2^ Centre for Maternal and Child Health Research, School of Health Sciences, City, University of London, London, United Kingdom

^3^ School of Anthropology, University of Oxford, Oxford, United Kingdom

^*^ Correspondence author

E-mail: [yangmei.li@npeu.ox.ac.uk](mailto:yangmei.li@npeu.ox.ac.uk) (YL)

**Supplementary Table 3 (S3 Table) The association between ethnic group and infant mortality (singleton live births, England and Wales, 2006-2012, full results for covariates in the adjusted models)**

|  | **Model A^a^** | | **Model B^a, b^** | |
| --- | --- | --- | --- | --- |
|  | OR | (95% CI) | OR | (95% CI) |
| **Infant’s ethnic group** |  |  |  |  |
| White British | 1 | - | 1 | - |
| Other White | 0.98 | (0.91-1.06) | 0.99 | (0.91-1.08) |
| Indian | 1.51 | (1.37-1.67) | 1.24 | (1.11-1.38) |
| Pakistani | 2.61 | (2.43-2.80) | 2.32 | (2.15-2.50) |
| Bangladeshi | 1.65 | (1.45-1.87) | 1.47 | (1.28-1.69) |
| Black Caribbean | 1.69 | (1.50-1.91) | 1.02 | (0.89-1.17) |
| Black African | 1.78 | (1.63-1.94) | 1.17 | (1.06-1.29) |
| Mixed/Other | 1.21 | (1.14-1.29) | 1.06 | (0.99-1.13) |
| Not stated | 1.25 | (1.17-1.33) | 1.12 | (1.04-1.20) |
| **Sex of infant** |  |  |  |  |
| Male | 1 | - | 1 | - |
| Female | 0.81 | (0.79-0.84) | 0.85 | (0.82-0.88) |
| **Infant’s year of birth** |  |  |  |  |
| 2006 | 1 | - | 1 | - |
| 2007 | 1.01 | (0.95-1.07) | 1.06 | (1.00-1.13) |
| 2008 | 0.92 | (0.87-0.98) | 0.96 | (0.90-1.02) |
| 2009 | 0.89 | (0.84-0.94) | 0.94 | (0.88-1.00) |
| 2010 | 0.84 | (0.79-0.89) | 0.92 | (0.87-0.98) |
| 2011 | 0.83 | (0.79-0.89) | 0.89 | (0.83-0.95) |
| 2012 | 0.75 | (0.71-0.80) | 0.82 | (0.77-0.88) |
| **Age of mother, years** |  |  |  |  |
| Under 18 | 1.51 | (1.36-1.68) | 1.41 | (1.25-1.58) |
| 18-19 | 1.37 | (1.27-1.48) | 1.45 | (1.34-1.58) |
| 20-24 | 1.12 | (1.06-1.17) | 1.22 | (1.16-1.29) |
| 25-29 | 1.03 | (0.98-1.08) | 1.08 | (1.03-1.13) |
| 30-34 | 1 | - | 1 | - |
| 35-39 | 1.09 | (1.03-1.15) | 0.99 | (0.93-1.04) |
| 40 and over | 1.43 | (1.32-1.55) | 1.23 | (1.13-1.35) |
| **Deprivation quintile** |  |  |  |  |
| 1 (most deprived) | 1.59 | (1.50-1.69) | 1.35 | (1.26-1.43) |
| 2 | 1.41 | (1.32-1.50) | 1.25 | (1.17-1.33) |
| 3 | 1.20 | (1.12-1.28) | 1.12 | (1.04-1.20) |
| 4 | 1.07 | (1.00-1.14) | 1.02 | (0.95-1.10) |
| 5 (least deprived) | 1 | - | 1 | - |
| **Mother's country of residence** | |  |  |  |
| England | 1 | - | 1 | - |
| Wales | 1.08 | (1.01-1.17) | 1.07 | (0.99-1.16) |
| **Mother’s country of birth** | |  |  |  |
| UK | 1 | - | 1 | - |
| Non-UK | 0.92 | (0.88-0.97) | 0.97 | (0.92-1.02) |
| **Marital status/registration type** | |  |  |  |
| Married | 1 | - | 1 | - |
| Joint registration/same address | 1.18 | (1.13-1.23) | 1.04 | (0.99-1.09) |
| Joint registration/different address | 1.42 | (1.34-1.50) | 1.07 | (1.00-1.13) |
| Sole registration | 1.46 | (1.37-1.56) | 1.15 | (1.08-1.24) |
| **Gestational age (completed weeks)** | |  |  |  |
| Extremely preterm (<28 weeks) | | | 457.10 | (436.06-479.16) |
| Very preterm (28-31 weeks) | | | 42.37 | (39.77-45.14) |
| Moderately preterm (32-33 weeks) | | | 15.44 | (14.15-16.85) |
| Late preterm (34-36 weeks) | | | 6.56 | (6.18-6.96) |
| Early term (37-38 weeks) | |  | 2.20 | (2.08-2.31) |
| Full term (39-41 weeks) | |  | 1 | - |
| Post-term (42 weeks) | |  | 1.04 | (0.91-1.20) |

^a^ Adjusted for sex of infant, infant’s year of birth, age of mother, deprivation quintile, mother's country of birth (UK vs. non-UK) and marital status/registration type

^b^ Additionally adjusted for gestational age in completed weeks (under 28, 28-31, 32-33, 34-36, 37-38, 39-41, 42)
