# Supplementary material for: The contribution of gestational age, area deprivation and mother’s country of birth to ethnic variations in infant mortality in England and Wales: A national cohort study using routinely collected data
Source: PLoS One. 2018 Apr 12;13(4):e0195146. doi: 10.1371/journal.pone.0195146 (PMC5896919; doi:10.1371/journal.pone.0195146)
Supplement: S4 Table — (DOCX) [file pone.0195146.s004.docx]

**Article title:**

## The contribution of gestational age, area deprivation and mother’s country of birth to ethnic variations in infant mortality in England and Wales: a national cohort study using routinely collected data

**Journal name:**

## Plos One

**Author names and affiliations:**

## Yangmei Li^1*^, Maria A. Quigley^1^, Nirupa Dattani^2^, Ron Gray^1^, Hiranthi Jayaweera^3^, Jennifer J. Kurinczuk^1^, Alison Macfarlane^2^, Jennifer Hollowell^1^

^1^ Policy Research Unit in Maternal Health and Care, National Perinatal Epidemiology Unit, Nuffield Department of Population Health, University of Oxford, Oxford, United Kingdom

^2^ Centre for Maternal and Child Health Research, School of Health Sciences, City, University of London, London, United Kingdom

^3^ School of Anthropology, University of Oxford, Oxford, United Kingdom

^*^ Correspondence author

E-mail: [yangmei.li@npeu.ox.ac.uk](mailto:yangmei.li@npeu.ox.ac.uk) (YL)

**Supplementary Table 4 (S4 Table) The association between ethnic group and infant mortality excluding congenital anomalies and stratified by gestational age (singleton live births, England and Wales, 2006-2012)**

| **Infant's ethnic group** | **Live births** | **Infant deaths** | **Infant mortality rate** | | **Model B^a, ,b, c^** | |
| --- | --- | --- | --- | --- | --- | --- |
|  | N | n | per 1,000 live births | (95% CI) | OR | (95% CI) |
| **Term infants^d^** |  |  |  |  |  |  |
| White British | 2,842,568 | 2,452 | 0.86 | (0.83-0.90) | 1 | - |
| Other White | 324,779 | 199 | 0.61 | (0.53-0.70) | 0.91 | (0.78-1.08) |
| Indian | 124,667 | 103 | 0.83 | (0.68-1.00) | 1.25 | (1.01-1.54) |
| Pakistani | 169,456 | 244 | 1.44 | (1.27-1.63) | 1.93 | (1.66-2.25) |
| Bangladeshi | 58,984 | 57 | 0.97 | (0.75-1.25) | 1.27 | (0.96-1.68) |
| Black Caribbean | 43,605 | 60 | 1.38 | (1.07-1.77) | 1.24 | (0.96-1.61) |
| Black African | 144,542 | 131 | 0.91 | (0.76-1.08) | 1.15 | (0.94-1.41) |
| Mixed/Other | 396,292 | 347 | 0.88 | (0.79-0.97) | 1.08 | (0.96-1.23) |
| Not stated | 271,524 | 252 | 0.93 | (0.82-1.05) | 1.11 | (0.97-1.27) |
| **Preterm infants^d^** |  |  |  |  |  |  |
| White British | 166,663 | 3947 | 23.68 | (22.96-24.42) | 1 | - |
| Other White | 15,747 | 376 | 23.88 | (21.61-26.38) | 1.09 | (0.95-1.25) |
| Indian | 7,984 | 182 | 22.80 | (19.74-26.31) | 0.96 | (0.81-1.15) |
| Pakistani | 10,813 | 385 | 35.61 | (32.27-39.27) | 1.38 | (1.20-1.58) |
| Bangladeshi | 3,964 | 90 | 22.70 | (18.50-27.84) | 1.08 | (0.84-1.38) |
| Black Caribbean | 3,900 | 174 | 44.62 | (38.57-51.56) | 0.98 | (0.81-1.17) |
| Black African | 9,534 | 445 | 46.68 | (42.62-51.10) | 1.07 | (0.92-1.23) |
| Mixed/Other | 23,678 | 645 | 27.24 | (25.24-29.39) | 0.96 | (0.86-1.06) |
| Not stated | 16,232 | 489 | 30.13 | (27.60-32.87) | 1.06 | (0.95-1.19) |

^a^ Adjusted for sex of infant and infant’s year of birth

^b^ Additionally adjusted for age of mother, deprivation quintile, mother's country of birth (UK vs. non-UK) and marital status/registration type

^c^ Additionally adjusted for gestational age in completed weeks (37-38, 39-41, 42 for term infants and under 28, 28-31, 32-33, 34-36 for preterm infants)

^d^ The interaction term between ethnic group and preterm birth was significant at the 1% level
